# The prevalence of metabolic syndrome in patients with non-alcoholic fatty liver disease in primary care clinics at King Saud University Medical City, Riyadh, Saudi Arabia

**DOI:** 10.3389/fendo.2025.1551201

**Published:** 2025-04-03

**Authors:** Alanoud Maan Alyousef, Doaa Zeinhom Mekawy, Yaser Yousef Bashumeel, Saleh Magdy Mohamed, Turky H. Almigbal, Mohammed A. Batais, Abdullah A. Alrasheed

**Affiliations:** ^1^ Department of Family Medicine, Johns Hopkins Aramco Hospital, Dhahran, Saudi Arabia; ^2^ Saudi Family Medicine Board, King Saud University Medical Hospital, Riyadh, Saudi Arabia; ^3^ Department of Anesthesia and Critical Care, Vision Colleges, Riyadh, Saudi Arabia; ^4^ Department of Anesthesia and Critical Care, Elsheikh Zayed Speciality Hospital, Cairo, Egypt; ^5^ Endocrine and Oncology Division, Department of Surgery, Tulane University School of Medicine, New Orleans, LA, United States; ^6^ Department of Family Medicine, Fal-Alafia Medical Complex, Riyadh, Saudi Arabia; ^7^ Department of Family and Community Medicine, College of Medicine, King Saud University, Riyadh, Saudi Arabia; ^8^ King Saud University Medical City, King Saud University, Riyadh, Saudi Arabia

**Keywords:** non-alcoholic fatty liver disease, metabolic syndrome, NCEP/ATP-III, hyperglycemia, Saudi Arabia

## Abstract

**Background:**

Metabolic syndrome is present in a subset of individuals harboring a constellation of metabolic risk factors that heightens their likelihood of developing coronary artery disease. Non-alcoholic fatty liver disease (NAFLD) manifests through the incremental accumulation of fat within liver cells in the absence of secondary causes. NAFLD has long been recognized as the hepatic manifestation of metabolic syndrome. Our study seeks to ascertain the prevalence of metabolic syndrome among NAFLD patients at King Khalid University Hospital and to explore the factors associated with metabolic syndrome.

**Method and design:**

We conducted a retrospective study targeting 1,173 patients diagnosed with NAFLD at King Khalid University Hospital in Riyadh, Saudi Arabia, from March 2020 to March 2021. NAFLD diagnosis was made based on ultrasonographic evidence of a fatty liver, excluding other liver ailments and alcohol intake. Metabolic syndrome was defined according to the National Cholesterol Education Program Adult Treatment Panel III (NCEP/ATP III) criteria, which require at least three of five metabolic risk factors to be present. Statistical analysis was performed using chi-square tests for categorical variables and independent t-tests for continuous variables, with a significance level set at p < 0.05.

**Results:**

Out of 1173 NAFLD participants evaluated, 38.2% met the NCEP/ATPIII criteria for metabolic syndrome. Additionally, 23.8% had at least one metabolic syndrome component coinciding with their ultrasonographically confirmed NAFLD diagnosis. The incidence of NAFLD was not linked to gender. Married individuals constituted a higher percentage (42.8%) of the NAFLD cohort. Elevated blood glucose and triglyceride levels, along with reduced HDL levels, were predominantly observed among the metabolic syndrome components in NAFLD patients.

**Conclusion:**

A significant portion of the NAFLD patient population was concurrently affected by metabolic syndrome. There exists a marked interrelationship between NAFLD and the components of metabolic syndrome. Regular metabolic disorder screenings are recommended for this patient group.

## Introduction

Non-alcoholic fatty liver disease (NAFLD) is a major cause of elevated liver enzymes and is increasingly recognized as the hepatic manifestation of metabolic syndrome (MetS) (1). It is characterized by excessive fat accumulation in hepatic cells, occurring independently of secondary causes such as alcohol abuse or viral hepatitis (2). A 2012 study in Jeddah, Saudi Arabia, estimated NAFLD prevalence at 16.6% (3). Although NAFLD often remains asymptomatic, it can progress to non-alcoholic steatohepatitis (NASH), fibrosis, cirrhosis, and even hepatocellular carcinoma in 20% of cases (4).

The global rise in NAFLD is closely linked to the increasing prevalence of metabolic risk factors, including obesity, diabetes, and dyslipidemia (5). In Saudi Arabia, obesity (35.5%), diabetes (23.7%), and dyslipidemia (54%) are highly prevalent (3). MetS—a cluster of abdominal obesity, insulin resistance, hypertension, and dyslipidemia—is a significant risk factor for NAFLD (6). The prevalence of MetS varies worldwide, influenced by genetic, lifestyle, and environmental factors, with Saudi Arabia reporting a prevalence of 39.8% using NCEP/ATP III criteria (7).

Despite the strong association between NAFLD and MetS, data on their combined prevalence in Saudi Arabia remains limited. Most studies have independently assessed NAFLD or MetS without exploring their coexistence. Given the rising metabolic disease burden in the region, understanding the prevalence of MetS among NAFLD patients and identifying the most frequently associated metabolic components is crucial. This study aims to fill this gap by assessing the prevalence and characteristics of MetS in NAFLD patients, providing epidemiological insights that could guide screening and management strategies in primary care settings (6–10).

## Materials and methods

### Study design and ethical considerations

This study was designed as a retrospective observational study using existing medical records of patients diagnosed with non-alcoholic fatty liver disease (NAFLD) at King Saud University Hospital in Riyadh, Saudi Arabia. The study period spanned from March 2020 to March 2021, and included data from patients diagnosed between 2016 and 2020. No direct patient recruitment, enrollment, or follow-up occurred. Given the increasing burden of metabolic disorders in Saudi Arabia, this timeframe remains highly relevant, as the prevalence and clinical implications of NAFLD/metabolic dysfunction-associated steatotic liver disease (MASLD) continue to be significant in both local and global populations.

The Institutional Ethical Review Board of the College of Medicine at King Saud University approved this study. Due to its retrospective nature, individual patient consent was not required in accordance with institutional guidelines. Strict measures were taken to ensure patient confidentiality throughout the study.

Recent guidelines have redefined NAFLD as metabolic dysfunction-associated steatotic liver disease (MASLD), reflecting its strong association with metabolic syndrome. While our study was conducted prior to this classification change, the underlying disease characteristics remain unchanged. Therefore, we have maintained the original term NAFLD throughout the manuscript to align with the historical data used in our study. However, we acknowledge this updated classification and have included it in the discussion as a recommendation for future research to apply MASLD criteria in epidemiological studies.

### Participant selection and screening

Participants of varying ages were included in the study, all of whom underwent fatty liver screening via ultrasonography (USG). Patients were selected for USG based on clinical indications, including abnormal liver function tests (elevated ALT, AST), obesity (BMI >30 kg/m²), type 2 diabetes mellitus, dyslipidemia, and metabolic syndrome risk factors. Additionally, some patients underwent screening as part of routine health checkups or comprehensive metabolic evaluations in primary care and endocrinology clinics.

Exclusion criteria were stringently applied to omit individuals with potential secondary causes of fatty liver, such as those taking hepatotoxic medications, with histories of alcohol consumption, viral hepatitis, autoimmune hepatitis, primary biliary cholangitis, or Wilson’s disease. Those who met the inclusion criteria were subsequently assessed for metabolic syndrome components according to the NCEP/ATPIII definition.

### Criteria for metabolic syndrome

The NCEP/ATPIII definition stipulates that metabolic syndrome is present when at least three of the following five criteria are met: a blood pressure higher than 130/85 mmHg, fasting triglyceride levels at or exceeding 150 mg/dL, fasting HDL cholesterol levels under 40 mg/dL for men or under 50 mg/dL for women, fasting blood sugar over 100 mg/dL or currently receiving treatment for elevated glucose, and waist circumference greater than 40 inches for men or 35 inches for women (11).

### Diagnosis of NAFLD

NAFLD diagnosis is typically confirmed using a combination of clinical, biochemical, and imaging assessments. While magnetic resonance imaging (MRI) and transient elastography (FibroScan) offer higher sensitivity in detecting mild hepatic steatosis, they are costly, less accessible, and not routinely available for large-scale screening in clinical practice.

For this study, abdominal ultrasonography (USG) was chosen as the primary diagnostic tool due to its wide availability, cost-effectiveness, and non-invasive nature. USG has been extensively validated in NAFLD research, demonstrating sensitivity between 60-94% and specificity between 84-95% for detecting hepatic steatosis. However, we acknowledge that its accuracy declines in patients with high BMI, and mild steatosis may not always be detected.

Given the retrospective nature of our study, we were limited to data from routine clinical evaluations, where USG is the standard first-line imaging modality. While MRI-PDFF and transient elastography offer greater precision, these modalities were not routinely performed in all patients due to logistical and financial constraints.

### Data management and statistical analysis

Data was systematically managed and coded using Microsoft Excel. Statistical analysis was conducted using SPSS^®^ version 26.0 (IBM Corporation, Armonk, NY, USA). To achieve the first objective (assessing the prevalence of metabolic syndrome in NAFLD patients), we performed descriptive statistical analysis, including frequency distributions and percentages for categorical variables, and means with standard deviations for continuous variables.

For the second objective (analyzing factors associated with metabolic syndrome in NAFLD patients), we used inferential statistical tests. Chi-square tests were applied to assess categorical variables such as gender, marital status, and smoking status. Independent t-tests were conducted to compare continuous variables, including fasting glucose, triglycerides, HDL cholesterol, blood pressure, and weight between groups with and without metabolic syndrome. Pearson correlation analysis was performed to evaluate associations between metabolic syndrome components and clinical parameters. Additionally, a stepwise multivariate logistic regression model was used to identify significant predictors of metabolic syndrome among NAFLD patients, with odds ratios (ORs) and 95% confidence intervals (CIs) reported. A p-value of <0.05 was considered statistically significant for all tests.

## Results

In this retrospective study, we analyzed 1247 participants diagnosed with non-alcoholic fatty liver disease (NAFLD) via ultrasonography (USG) at outpatient clinics. Of the participants, 46.7% were male, and 53.3% were female, with the majority (87.6%) being Saudi citizens. Marital status data showed 67% married and 33% single, while 96.4% were non-smokers. Alcohol consumption was reported in only 0.6% of participants, and a mere 1% had a positive family history of liver conditions (**Table 1**).

**Table 1:** Demographic and clinical characteristics of NAFLD patients in relation to metabolic syndrome.

FACTOR	LEVEL	FREQUENCY	PERCENT
GENDER	Male	582	46.7
	Female	664	53.3
Nationality	Non Saudi	154	12.4
	Saudi	1092	87.6
Marital status	Single	411	33.0
	Married	834	67.0
Smoker	No	1195	96.4
	Yes	45	3.6
Alcohol consumption	No	1231	99.4
	Yes	8	0.6
Family Hx	No	1230	99.0
	Yes	12	1.0
Met Syndrome	No	764	61.7
	Yes	474	38.2

Following the application of exclusion criteria, 1173 participants remained for inclusion in the study. Among these, 448 (38.2%) met the criteria for metabolic syndrome, while 725 (61.8%) did not (**Table 1**). A chi-square test revealed no significant association between the presence of NAFLD and factors such as gender, nationality, smoking status, or family history. However, marital status was significantly associated with NAFLD, with married participants showing a higher prevalence (42.8%) compared to singles (28.7%) (**Table 2**).

**Table 2:** Prevalence of metabolic syndrome among NAFLD patients based on demographic and lifestyle factors.

Factor	Level	Metabolic Syndrome						P value
		No		Yes		Total		
		N	Percent	N	Percent	N	Percent	
Gender	Male	328	61.2	208	38.8	536	100	0.707
	Female	396	62.3	240	37.7	636	100	
Nationality	Non-Saudi	96	67.6	46	32.4	142	100	0.133
	Saudi	629	61.1	401	38.9	1030	100	
Marital Status	Single	278	71.3	112	28.7	390	100	0.000*
	Married	447	57.2	334	42.8	781	100	
Smoker	No	699	62.2	425	37.8	1124	100	0.111
	Yes	21	50	21	50	42	100	
Family History	No	714	61.7	444	38.3	1158	100	0.589
	Yes	7	70	3	30	10	100	

*Statistically Significant at (α≤0.05).

Comparative analyses through an independent t-test indicated that individuals with NAFLD had statistically significant higher mean ages and measurements for fasting glucose, triglycerides, systolic and diastolic blood pressure, and weight than those without the disease. Notably, the mean fasting glucose for those with NAFLD was significantly higher than those without (**Table 3**).

**Table 3:** Comparison of clinical variables by metabolic syndrome status using independent samples t-test.

Metabolic Syndrome		N	Mean	Std. deviation	P-value
Age	No	725	42.23	14.645	0.000
	Yes	448	48.47	14.536	
BMI	No	708	37.24	121.415	0.797
	Yes	445	35.76	8.394	
Fasting Glucose	No	417	5.46	1.684	0.005
	Yes	328	9.40	28.794	
Triglycerides	No	675	1.46	3.021	0.000
	Yes	445	2.13	1.275	
HDL	No	674	1.60	5.966	0.555
	Yes	445	1.40	4.559	
Systolic Blood Pressure	No	721	129.10	18.268	0.000
	Yes	447	144.09	16.673	
Diastolic Blood Pressure	No	719	75.13	11.721	0.000
	Yes	446	79.88	12.428	
Height	No	699	162.72	15.275	0.961
	Yes	438	162.77	13.299	
Weight	No	714	87.70	24.326	0.000
	Yes	441	95.63	24.045	

*Statistically Significant at (α≤0.05).

Pearson correlation analysis revealed significant associations between metabolic syndrome and various clinical parameters. A positive correlation was observed between metabolic syndrome and age (r = 0.204, p < 0.01), indicating that older participants were more likely to have metabolic syndrome. Similarly, fasting glucose (r = 0.102, p < 0.05) and triglyceride levels (r = 0.133, p < 0.01) were significantly associated with metabolic syndrome, suggesting a strong link between hyperglycemia, dyslipidemia, and metabolic syndrome presence. Additionally, metabolic syndrome showed a strong positive correlation with systolic blood pressure (r = 0.381, p < 0.01) and a moderate correlation with diastolic blood pressure (r = 0.189, p < 0.01), reinforcing the connection between hypertension and metabolic syndrome. However, no significant correlation was found between metabolic syndrome and BMI (r = -0.008, p = 0.797) or HDL cholesterol levels (r = -0.018, p = 0.555) (**Table 4**).

**Table 4:** Correlation between metabolic syndrome components and various clinical parameters.

	AGE	BMI	Fasting glucose	Triglyceride	HDL	Systolic BP	Diastolic BP
Metabolic Syndrome							
Pearson Correlation	.204**	-0.008	.102*	.133**	-0.018	.381**	.189**
Sig. (2-tailed)	0.000	0.797	0.005	0.000	0.555	0.000	0.000
N	1173	1153	745	1120	1119	1168	1165

**. Correlation is significant at the 0.01 level (2-tailed). *. Correlation is significant at the 0.05 level (2-tailed).

The study further evaluated the frequency of metabolic syndrome components among participants. It was found that 5.4% of participants did not meet any criteria for metabolic syndrome, 23.8% met one criterion, 30.7% met two, 24.4% met three, 13.1% met four, and only 2.6% satisfied all five criteria of metabolic syndrome.

In **Table 5**, a stepwise multivariate logistic regression model is presented, analyzing the impact of various factors on an outcome variable. The factors included are fasting glucose, triglyceride levels, and systolic blood pressure (Systolic BP), along with a constant term.

**Table 5:** Stepwise multivariate logistic regression model.

Factor	B	SE	Wald df	Sig.	Exp(B) OR	95% CI for EXP(B)	
						Lower	Upper
Fasting Glucose	0.489	0.058	70.62	0.000	1.630	1.454	1.827
Triglyceride	1.434	0.160	79.92	0.000	4.193	3.062	5.742
Systolic BP	0.063	0.007	79.21	0.000	1.065	1.050	1.080
Constant	14.333	1.200	142.61	0.000	0.000		

For fasting glucose, the coefficient (B) is 0.489 with a standard error (SE) of 0.058. This factor is statistically significant as indicated by a Wald statistic of 70.62 degrees of freedom and a p-value (Sig.) of less than 0.001 (reported as 0.000). The odds ratio (Exp(B) OR) is 1.630, suggesting that one unit increase in fasting glucose is associated with a 63% increase in the odds of the outcome occurring. The 95% confidence interval for this odds ratio ranges from 1.454 to 1.827, indicating precision and reliability in the estimate.

The triglyceride level has a coefficient of 1.434, a standard error of 0.160, and is also statistically significant with a Wald statistic of 79.92 degrees of freedom and a p-value of less than 0.001. The odds ratio for triglycerides is substantially higher at 4.193, meaning that a one unit increase in triglyceride level is associated with over a fourfold increase in the odds of the outcome. The confidence interval for this estimate is wide but still indicates a strong effect, ranging from 3.062 to 5.742.

Systolic BP’s coefficient is 0.063 with a very small standard error of 0.007. It is statistically significant (p-value < 0.001) with a Wald statistic of 79.21 degrees of freedom. The corresponding odds ratio is 1.065, implying a more modest increase in the odds of the outcome with each unit increase of systolic blood pressure. The 95% confidence interval for the odds ratio is tight, from 1.050 to 1.080, suggesting a consistent effect across the sample.

Lastly, the model includes a constant term with a coefficient of 14.333 and a standard error of 1.200. It is highly significant (p-value < 0.001) with a Wald statistic of 142.61 degrees of freedom. The odds ratio for the constant is reported as 0.000, but this is typically a placeholder value in statistical output indicating that the odds ratio for the constant is not usually interpreted.

This data underscores the substantial overlap between NAFLD and metabolic syndrome components, indicating a potential interplay between these health issues within the studied population.

## Discussion

The findings of this study bring to light the significant association between non-alcoholic fatty liver disease (NAFLD) and metabolic syndrome, with nearly two-fifths of the NAFLD patients exhibiting the concurrent presence of metabolic syndrome as defined by NCEP/ATPIII criteria. This high prevalence underscores the intricate link between hepatic steatosis and metabolic disturbances (11–13).

The variance in prevalence rates of metabolic syndrome in NAFLD patients between this study and that conducted in Nepal is an important observation, highlighting the potential influence of regional, ethnic, and methodological differences on the manifestation of these conditions. Factors such as diet, lifestyle, genetic predisposition, and even the criteria used to define metabolic syndrome may contribute to such discrepancies and warrant further exploration.

Our findings indicate that 38.2% of NAFLD patients met the criteria for metabolic syndrome, aligning with the growing body of evidence that supports a strong link between hepatic steatosis and metabolic disturbances. This prevalence is comparable to a study conducted in Saudi Arabia, which reported a metabolic syndrome prevalence of 39.8% among NAFLD patients (4).

Internationally, our results are slightly higher than those reported in a Nepalese study (9), which found a metabolic syndrome prevalence of 34.8% among NAFLD patients. Differences in dietary patterns, genetic predisposition, and lifestyle factors may contribute to this variation. Additionally, our findings are lower than those reported in an Indian study (12), where the prevalence exceeded 45%, suggesting a higher metabolic risk burden in South Asian populations.

In Western populations, the prevalence of metabolic syndrome among NAFLD patients varies. A study from the United States (8) found that nearly 50% of NAFLD patients met metabolic syndrome criteria, highlighting potential differences in obesity rates, sedentary lifestyles, and dietary factors between Middle Eastern and Western populations. Furthermore, a European study by Marchesini et al. (10) reported a prevalence of 41%, slightly higher than our findings but within a comparable range. These variations emphasize the importance of region-specific metabolic risk profiles and screening strategies.

Our study further aligns with findings from Al-Hamoudi et al. (6), who identified dyslipidemia, hypertension, and hyperglycemia as the most prevalent metabolic syndrome components in Saudi NAFLD patients. This consistency supports the hypothesis that insulin resistance serves as a common pathway linking NAFLD and metabolic syndrome across different populations.

These regional differences highlight the need for customized screening and prevention strategies based on local risk factor distributions. Future multi-center studies could provide a more comprehensive understanding of the metabolic burden among NAFLD patients across different ethnic groups.

The advanced age of participants with metabolic syndrome compared to their counterparts suggests a cumulative effect of metabolic risk factors over time leading to both metabolic syndrome and NAFLD. This age-related trend points towards the importance of early intervention in metabolic risk factors to potentially prevent the development of NAFLD (12).

Gender differences in the prevalence of metabolic syndrome amongst the NAFLD cohort did not align with data from Nepal, indicating that the relationship between gender, NAFLD, and metabolic syndrome may be more complex than previously thought. While hormonal factors are traditionally considered influential, this study’s findings suggest that other factors such as lifestyle or genetic background may play a more prominent role in this cohort (12–15).

The associations between NAFLD and traditional components of metabolic syndrome such as dysglycemia, dyslipidemia, and hypertension were also evident in this study, reinforcing the concept of NAFLD as a hepatic manifestation of metabolic syndrome. The high prevalence of these risk factors in the NAFLD population suggests a shared pathophysiological pathway that may involve insulin resistance as a common denominator.

Interestingly, no association was found between smoking and metabolic syndrome within the NAFLD patients. This is in contrast with other studies and may reflect varying patterns of smoking behavior or possibly the interplay of other more dominant risk factors within this population. However, this result should be interpreted with caution, as the cross-sectional nature of this study limits the ability to establish causation.

The findings of this study underscore the strong association between NAFLD and metabolic syndrome, emphasizing the need for early detection and intervention in primary care settings. Given that **38.2%** of NAFLD patients met the criteria for metabolic syndrome, primary care physicians should consider routine screening for hypertension, dyslipidemia, insulin resistance, and obesity in this population¹. Current guidelines recommend that patients with NAFLD, particularly those with metabolic syndrome, undergo regular monitoring for liver fibrosis progression using non-invasive methods such as transient elastography (FibroScan) or fibrosis scores (FIB-4, NAFLD fibrosis score)². This is critical, as patients with NAFLD and metabolic syndrome are at a significantly higher risk for advanced liver disease and cardiovascular complications³. Integrating metabolic risk assessments into routine primary care visits could enhance early detection and facilitate targeted interventions aimed at reducing morbidity and mortality in this high-risk group.

The results also suggest that addressing modifiable metabolic risk factors could reduce the burden of NAFLD-related complications. Lifestyle interventions, particularly weight loss of 5-10% of body weight, have been shown to improve hepatic steatosis and metabolic parameters (4). This should be encouraged through a combination of dietary modifications (such as adherence to a Mediterranean or low-carbohydrate diet) and regular physical activity (at least 150 minutes per week of moderate-intensity exercise) (5). While no FDA-approved pharmacologic treatments exist for NAFLD, certain agents such as metformin, GLP-1 receptor agonists, and SGLT-2 inhibitors have shown promise in improving metabolic parameters in patients with coexisting NAFLD and type 2 diabetes (6). Moreover, despite concerns about hepatotoxicity, statins can be safely used in NAFLD patients with dyslipidemia (7). Given the heightened cardiovascular risk among NAFLD patients with metabolic syndrome, regular lipid profiling, blood pressure monitoring, and cardiovascular risk stratification should be incorporated into standard care (8). These findings highlight the need for primary care physicians to adopt a proactive, multidisciplinary approach in managing NAFLD and metabolic syndrome, while future randomized clinical trials should explore the long-term benefits of pharmacologic interventions and intensive lifestyle modifications in this high-risk population (9, 10).

The limitations of this study, particularly the retrospective design, precluded certain measurements and assessments, such as waist circumference, which is a key component of metabolic syndrome. The inability to assess genetic factors and perform liver fibrosis staging also limits the depth of understanding regarding the severity of NAFLD and its interplay with metabolic syndrome. This gap points to the need for retrospective studies with comprehensive data collection, including genetic profiling and advanced liver imaging techniques, to fully characterize the relationship between NAFLD and metabolic syndrome. Furthermore, longitudinal studies could provide valuable insights into the temporal sequence of events and causality, contributing to the development of targeted prevention and treatment strategies for this patient population.

This study utilized ultrasonography (USG) as the primary diagnostic tool for NAFLD due to its non-invasive nature, cost-effectiveness, and accessibility in routine clinical practice. However, it is important to acknowledge that USG has limitations in detecting mild hepatic steatosis, particularly when fat accumulation is below 20% of hepatocytes. Studies have demonstrated that MRI-based techniques, such as proton density fat fraction (MRI-PDFF), have higher sensitivity and specificity in detecting early-stage NAFLD and quantifying hepatic fat content. Consequently, our study may have underestimated the true prevalence of NAFLD, particularly in individuals with mild disease. Additionally, USG is operator-dependent, and subtle differences in technique and interpretation may introduce variability in diagnostic accuracy. Despite these limitations, USG remains a widely accepted and practical tool for large-scale epidemiological studies and routine clinical assessments. Future studies incorporating MRI or transient elastography (FibroScan) may provide a more precise estimation of NAFLD prevalence and severity.

The exclusion of patients with secondary causes of fatty liver, such as alcohol consumption, viral hepatitis, autoimmune hepatitis, and other chronic liver diseases, ensures that the findings specifically reflect the relationship between NAFLD and metabolic syndrome. However, these exclusions may limit the generalizability of our results to broader populations where mixed etiologies of liver disease are more prevalent. In clinical settings where alcohol-related liver disease or viral hepatitis is common, the overlap between these conditions and metabolic syndrome could alter the observed prevalence and associations. Additionally, our exclusion criteria may underestimate the real-world burden of metabolic dysfunction-associated liver disease (MAFLD), a broader category that includes individuals with multiple metabolic risk factors, even in the presence of other liver diseases. Future studies should explore these relationships in more diverse patient populations to better understand the full spectrum of metabolic and hepatic disorders.

Another potential limitation of our study is the possibility of selection bias. Patients who underwent ultrasonography may have been those with higher metabolic risk factors, abnormal liver function tests, or clinical suspicion of NAFLD, leading to a sample that overrepresents individuals with more severe metabolic profiles. This could influence the estimated prevalence of metabolic syndrome within our NAFLD cohort. Moreover, patients with milder or asymptomatic NAFLD who were not referred for ultrasonography may have been underrepresented in our study. Future studies should consider population-based screening approaches or utilize broader inclusion criteria to capture a more representative sample of NAFLD patients across different severity levels.

## Conclusion and future directions

This study highlights the high prevalence of metabolic syndrome (38.2%) among NAFLD patients and underscores the strong association between NAFLD and key metabolic risk factors, including hyperglycemia, hypertension, and dyslipidemia. The findings suggest that routine screening for metabolic syndrome in NAFLD patients should be a priority in clinical practice to identify high-risk individuals and implement early interventions. Given the increasing burden of metabolic disorders in Saudi Arabia, targeted lifestyle and pharmacologic interventions may help reduce disease progression and improve long-term health outcomes.

Future research should focus on prospective, longitudinal studies to establish causal relationships between NAFLD and metabolic syndrome components, as well as to assess the impact of specific interventions on disease progression. Additionally, genetic profiling and advanced imaging techniques, such as MRI-PDFF and transient elastography, should be incorporated into future studies to provide a more accurate assessment of NAFLD severity and metabolic risk factors. Expanding research to include diverse populations and exploring the potential role of novel pharmacologic agents in NAFLD management will further enhance our understanding of the disease and its implications for public health.

## Data Availability

The raw data supporting the conclusions of this article will be made available by the authors, without undue reservation.
